# The Impact of Bulbar and Upper Motor Neuron Involvement on Oculomotor Movement in Amyotrophic Lateral Sclerosis

**DOI:** 10.1002/brb3.70906

**Published:** 2025-10-23

**Authors:** Dongchao Shen, Anfeng Liu, Xunzhe Yang, Qing Liu, Mingsheng Liu, Liying Cui

**Affiliations:** ^1^ Department of Neurology Peking Union Medical College Hospital Beijing China; ^2^ Chinese Academy of Sciences—Ruiyi Information Technology Beijing China; ^3^ Neuroscience Center Chinese Academy of Medical Sciences and Peking Union Medical College Beijing China

**Keywords:** amyotrophic lateral sclerosis, bulbar involvement, oculomotor function, onset, upper motor neuron

## Abstract

**Background:**

Amyotrophic lateral sclerosis (ALS) is a progressive neurodegenerative disease characterized by the degeneration of both lower and upper motor neurons (UMN). Clinical heterogeneity manifests in subtypes such as bulbar‐onset ALS (bALS) and spinal‐onset ALS (sALS), with emerging evidence suggesting that oculomotor dysfunction may reflect broader multisystem involvement. This study aims to investigate oculomotor parameters across different ALS phenotypes and their associations with neuropsychological domains.

**Methods:**

A total of 46 patients meeting the Gold Coast Criteria for ALS were enrolled, alongside 23 age‐ and education‐matched healthy controls (HCs). Participants were assessed for demographic variables and clinical features, and underwent cognitive and oculomotor testing using the EyeKnow system. Eye movement performance was compared between groups, and correlations between oculomotor metrics and cognitive and clinical data were examined.

**Results:**

ALS patients displayed longer reaction times in anti‐saccade tasks (357.48 ± 61.28 ms vs. 316.10 ± 52.70 ms, *p* = 0.005) and significantly lower predictive saccade accuracy (86.77 ± 19.17% vs. 99.36 ± 2.22%, *p* < 0.001) compared to HCs. There is no significant difference in the eye movement parameters between sALS and bALS. Patients with bulbar involvement exhibited poorer performance in predictive saccade accuracy (77.53 ± 26.66% vs. 96.01 ± 4.92%, *p* < 0.001) and longer initial time in the smooth pursuit task (647.43 [402.14, 760.64] ms vs. 452.43 [131.62, 598.20] ms, *U* = 161.00, *p* = 0.037) compared to those without bulbar involvement. UMN involvement was associated with poorer performance across prosaccade, anti‐saccade, and predictive saccade tasks. No significant correlation between oculomotor metrics and cognitive tests or clinical data was detected.

**Conclusions:**

The findings highlight the impact of bulbar and UMN involvement on oculomotor dysfunction in ALS, demonstrating distinct patterns across various phenotypes. Although oculomotor metrics show sensitivity to the pathophysiology of ALS, their effectiveness as independent biomarkers needs further validation through longitudinal studies that include larger cohorts, advanced neuroimaging techniques, and multimodal assessments to capture the complex interplay between motor, cognitive, and anatomical changes in this varied disease.

## Graphical abstract

Oculomotor assessment revealed that ALS patients, especially those with bulbar or upper motor neuron (UMN) involvement, showed slower anti‐saccade reaction times and lower predictive saccade accuracy than healthy controls. These findings suggest distinct patterns of oculomotor dysfunction among ALS subgroups, highlighting the value of these metrics for ALS phenotyping.

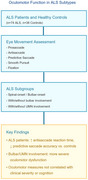

## 1 Introduction

Amyotrophic lateral sclerosis (ALS) is a progressive neurodegenerative disease characterized by the degeneration of both upper (UMN) and lower motor neurons (LMN). Clinical heterogeneity is evident in its subtypes: bulbar‐onset ALS (bALS), which accounts for approximately 20%–30% of cases, involves earlier and more extensive brainstem pathology, typically resulting in pronounced dysarthria and dysphagia, whereas spinal‐onset ALS (sALS) initially presents with limb weakness before progressing to involve other motor systems (Feldman et al. 2022; Goutman et al. 2022). UMN involvement further diversifies phenotypes and is generally associated with spasticity, hyperreflexia, and pseudobulbar symptoms. These different clinical phenotypes reflect the heterogeneity of ALS and underscore the importance of determining how distinct patterns of neuronal involvement might shape disease progression and its biomarkers.

In recent years, ALS has been increasingly recognized as a multisystem disorder, with accumulating evidence of extramotor involvement, including cognitive and behavioral dysfunctions that overlap with frontotemporal dementia (FTD). Among emerging biomarkers, oculomotor dysfunction has gained attention for its dual role in reflecting both motor and cognitive decline. Oculomotor control relies on the integration of cortical (e.g., frontal eye fields [FEF]), subcortical (e.g., basal ganglia), and brainstem networks (Lynch and Tian 2006), making it sensitive to ALS‐related neurodegeneration and potentially serving as a window into the multisystem nature of ALS.

While emerging evidence suggests that bulbar involvement and UMN pathology may differentially influence oculomotor parameters in ALS, the literature remains inconsistent regarding these associations. In bALS, studies indicate slower saccades, more anti‐saccade errors, and reduced smooth pursuit gains than in sALS (Donaghy et al. 2010; Proudfoot et al. 2015; Kang et al. 2018), likely due to early brainstem degeneration and impaired fronto‐brainstem connectivity (Zaino et al. 2022). Conversely, other investigations detect no significant differences in eye movement parameters between these subtypes (Burrell et al. 2013; Guo et al. 2022). Similarly, conflicting results exist for UMN‐related oculomotor dysfunction: although phenotypes with prominent UMN signs demonstrate higher rates of oculomotor abnormalities compared to classic ALS or predominantly LMN diseases (Poletti et al. 2021), these correlations are not consistently replicated in other cohorts (Youn et al. 2023). Such discrepancies may arise from methodological variability (e.g., eye‐tracking protocols, UMN burden quantification) or confounding factors like disease progression heterogeneity and cognitive decline, highlighting the need for standardized assessments to disentangle the contributions of bulbar and UMN pathology to oculomotor dysfunction in ALS.

A more comprehensive understanding of these relationships may illuminate the underlying pathophysiological mechanisms, help refine disease phenotyping, and advance the development of noninvasive oculomotor metrics as biomarkers for disease progression and clinical decision‐making. In the present study, we characterized oculomotor parameters across different ALS phenotypes and their associations with neuropsychological domains, addressing gaps and variations in the literature to inform both clinical care and future research.

## 2 Methods

A total of 46 patients who met the Gold Coast Criteria (Shefner et al. 2020) for ALS were enrolled in this study. All patients underwent electromyography (EMG) examination of the four body regions (bulbar, cervical, thoracic, and lumbosacral). Additionally, 23 age‐ and education‐matched healthy controls (HCs) were included. Participants were excluded if they had neurological conditions affecting cognition (e.g., other neurodegenerative diseases, major stroke, traumatic head injuries, or severe psychiatric illness), a history of eye or vestibular disorders impacting eye movements, or were taking medications known to influence eye movements, such as diazepam, clonazepam, or antipsychotics.

Demographic and clinical data were systematically collected. Disease duration was defined as the interval between the onset of initial symptoms and the date of oculomotor assessment. Disease severity was assessed using the revised ALS Functional Rating Scale (ALSFRS‐R). The bulbar score was calculated as the sum of the speech, salivation, and swallowing subscores. The rate of disease progression was determined using the formula: (48—ALSFRS‐R score) divided by disease duration (in months). ALS patients were categorized into subgroups based on three criteria for comparative analysis: (1) pattern of disease onset, (2) presence or absence of bulbar involvement, and (3) presence or absence of UMN involvement. The pattern of disease onset was classified as either bulbar or spinal. Bulbar involvement was characterized by symptoms such as dysarthria, dysphagia, or pseudobulbar features (e.g., involuntary crying or laughing). In this study, we did not distinguish between bulbar and corticobulbar involvement. Patients presenting only with positive jaw jerks, an exaggerated gag reflex, or EMG abnormalities in bulbar motor neuron‐innervated muscles (primarily the sternocleidomastoid in our laboratory), without accompanying clinical symptoms, were not categorized as having bulbar involvement. UMN dysfunction was defined by the presence of one or more of the following clinical signs: increased deep tendon reflexes, pathological reflexes (such as Babinski or Hoffman signs), spasticity (i.e., increased velocity‐dependent muscle tone), or slowed and poorly coordinated voluntary movements that could not be attributed to LMN or Parkinsonian causes. UMN burden was assessed using a clinical scale that quantified the extent, strength, and distribution of UMN involvement across different body regions, with a scoring range of 0–36 points (Mezzapesa et al. 2013). All participants completed cognitive assessments, including the mini‐mental state examination (MMSE), Montreal cognitive assessment (MOCA), frontal assessment battery (FAB), and Chinese version of the Edinburgh Cognitive and Behavioral ALS Screen (ECAS) (Ye et al. 2016).

Eye movements were monitored using the EyeKnow system (CAS‐Ruiyi Information Technology Co. Ltd., Beijing, China) with a sampling frequency of 120 Hz (Lin et al. 2024). The system comprises a tablet‐based console and a virtual reality (VR) eye tracker equipped with an integrated eye‐tracking camera. The VR eye tracker, designed for guiding and recording eye movements, has a compact cuboid design and occupies less than one square meter of space. A high‐resolution display (3664 × 1920 pixels) is built into the tracker and positioned near the participant's eyes, simulating a viewing distance of one meter for stimulus presentation. The system also includes an internal data processing module to analyze eye‐tracking parameters. A five‐point calibration process was performed at the start of each session, maintaining a maximum calibration error within a 2° radius. Seven tasks were performed, including pro‐saccade, anti‐saccade, predictive saccade, smooth pursuit, and fixation tasks (the procedures of eye‐tracking tasks and definition of oculomotor parameters are described in the Supporting Information). To ensure accuracy and objectivity, the paradigm may be retaken if any misunderstanding of the subjects for the task requirements was observed.

Statistical analyses were performed as follows: categorical data were expressed as percentages, while continuous measurements were reported as means ± standard deviations (SDs) or medians with interquartile ranges (IQRs), depending on their distribution patterns. Between‐group comparisons of demographic characteristics, gait parameters, and oculomotor indices were conducted using *χ* ^2^ tests for categorical data and either two‐sample *t*‐tests or Mann–Whitney tests for continuous parameters based on normality assumptions. Correlation analyses were conducted to examine relationships between oculomotor variables, neuropsychological assessments, and clinical measures, using Pearson's coefficient for normally distributed data and Spearman's rank correlation coefficient otherwise. Multiple linear regression was applied to assess associations between continuous variables while accounting for covariates. A two‐tailed *p* < 0.05 was considered indicative of statistical significance.

## 3 Results

The demographic and clinical characteristics of ALS patients and HC are summarized in Table 1. ALS patients and HCs showed no significant differences in age, sex distribution, or education level. Demographic data and clinical information were similar between sALS and bALS, patients with or without bulbar involvement, and patients with or without UMN involvement. Cognitive assessments showed no significant differences between ALS patients and HCs, although ALS patients exhibited lower cognitive performance on the ECAS, which did not reach statistical significance (*T* = 1.303, *p* = 0.197).

**TABLE 1:** Demographic, clinical and cognitive characteristics of ALS patients and healthy controls.

	sALS (*n* = 31)	bALS (*n* = 15)	ALS (*n* = 46)	Healthy controls (*n* = 23)	*p* value
Age, years	55.23 ± 9.10	56.58 ± 10.84	55.67 ± 9.58	56.17 ± 10.37	0.847
Male/female	14/17	6/9	20/26	12/11	
Education, years	9.86 ± 4.31	9.24 ± 3.78	9.70 ± 4.15	11.00 ± 4.13	0.225
Disease duration, months	20.74 ± 14.06	19.54 ± 9.13	20.43 ± 12.87		
Bulbar involvement	8	15	23		
UMN involvement	23	11	34		
UMN scores	12.29 ± 5.70	12.47 ± 4.89	12.35 ± 5.39		
ALSFRS‐R total score	42.00 (35.00, 44.00)	42.00 (38.00, 44.00)	42.00 (35.00, 44.00)		
ALSFRS‐R bulbar subscore	12.00 (10.00, 12.00)	8.00 (6.50, 10.00)	11.00 (8.23, 12.00)		
Disease progression rate	0.31 (0.19, 0.67)	0.50 (0.30, 1.00)	0.37 (0.22, 0.80)		
MMSE	26.68 ± 5.01	27.66 ± 3.29	27.00 ± 4.48	28.22 ± 1.86	0.116
MoCA	23.31 ± 5.78	21.13 ± 7.02	22.60 ± 6.13	24.00 ± 4.09	0.264
FAB	12.92 ± 4.92	12.77 ± 4.50	12.87 ± 4.74	13.43 ± 2.76	0.538
ECAS	89.05 ± 33.49	86.21 ± 32.18	88.13 ± 32.71	96.25 ± 18.91	0.197
ECAS‐ALS specific	66.10 ± 26.59	63.32 ± 24.51	65.20 ± 25.66	69.53 ± 14.89	0.380
ECAS‐non ALS specific	25.44 ± 7.83	23.88 ± 8.47	24.93 ± 7.95	26.63 ± 4.72	0.271

Abbreviations: ALS, amyotrophic lateral sclerosis; ALSFRS‐R, revised ALS Functional Rating Scale; ECAS, Edinburgh Cognitive and Behavioral ALS Screen (ECAS); FAB, frontal assessment battery; MMSE, mini‐mental state examination; MOCA, Montreal cognitive assessment; UMN, upper motor neuron.

### 3.1 Comparison Between ALS Patients and HCs

In the eye movement tasks, ALS patients demonstrated longer reaction time in the anti‐saccade task (357.48 ± 61.28 ms vs. 316.10 ± 52.70 ms, *T* = 2.909, *p* = 0.005) (Table 2). Additionally, ALS patients showed significant impairment in predictive saccade performance compared to HCs, with lower accuracy (86.77 ± 19.17% vs. 99.36 ± 2.22%, *T* = 4.396, *p* < 0.001). Other parameters in prosaccade, smooth pursuit, and fixation tasks showed no significant differences between groups, but there was a trend towards poorer performance in the ALS group.

**TABLE 2:** Comparison of oculomotor performance between ALS patients and healthy controls.

Task	Feature	ALS (*n* = 46)	Healthy controls (*n* = 23)	*p* value
Prosaccade	Accuracy, %	96.52 ± 10.68	98.60 ± 3.70	0.240
	Latency, ms	244.84 ± 43.61	236.13 ± 49.68	0.479
	Reaction time, ms	305.30 (269.61, 341.15)	324.22 (299.84, 391.39)	0.088
	Velocity, °/s	227.87 ± 83.81	241.97 ± 100.60	0.566
Anti‐saccade	Accuracy, %	42.86 (6.25, 57.14)	52.78 (9.38, 66.67)	0.431
	Latency, ms	343.37 ± 70.39	310.46 ± 61.75	0.061
	Reaction time, ms	357.48 ± 61.28	316.10 ± 52.70	**0.005**
	Velocity, °/s	223.52 ± 83.70	240.74 ± 75.60	0.394
	Uncorrected error rate, %	0.00 (0.00, 23.61)	0.00 (0.00, 25.00)	0.925
	Error correction reaction time, ms	296.85 ± 161.34	239.32 ± 178.45	0.200
Predictive saccade	Accuracy, %	86.77 ± 19.17	99.36 ± 2.22	**< 0.001**
	Latency, ms	335.27 (299.83, 370.15)	341.85 (314.63, 385.58)	0.443
	Number of predications	1.00 (0.00, 1.25)	0.00 (0.00, 0.75)	0.160
Smooth pursuit	Initial time, ms	530.85 (299.81, 681.34)	455.47 (286.83, 565.78)	0.410
	Tracking velocity, °/s	21.86 (15.44, 29.25)	19.21 (13.41, 25.18)	0.547
	Tracking acceleration, °/s^2^	32.57 (21.12, 103.01)	38.33 (25.71, 88.17)	0.650
	Number of deviations	14.00 (9.00, 27.00)	20.00 (9.00, 33.00)	0.496
	Deviation (> 4°), °	5.25 (4.86, 5.25)	5.17 (4.91, 5.79)	0.795
	Total deviation (> 4°), °	78.74 (54.48, 141.99)	98.75 (52.13, 178.11)	0.480
Fixation	Accuracy, %	82.04 ± 24.93	83.50 ± 16.61	0.773
	Number of deviations (> 4°)	6.00 (2.00, 23.00)	8.00 (2.00, 21.00)	0.927
	Number of deviations (> 2°)	18.00 (4.00, 38.50)	25.00 (14.00, 43.00)	0.150
	Total deviation (> 4°), °	35.36 (17.05, 125.90)	58.51 (12.98, 138.93)	0.953
	Total deviation duration, ms	665.34 (377.19, 2152.34)	1109.56 (410.00, 2416.43)	0.564

Abbreviation: ALS, amyotrophic lateral sclerosis. Bold values indicate statistically significant results.

### 3.2 Comparison Between sALS and bALS Patients

When comparing sALS and bALS patients, no differences were observed across all eye movement tasks (Table 3). Both subgroups showed significantly prolonged reaction time in anti‐saccade (*T* = 2.483, *p* = 0.016 and *T* = 2.626, *p* = 0.013, respectively) and reduced accuracy in predictive saccade (*T* = 2.849, *p* = 0.008 and *T* = 3.913, *p* = 0.002, respectively) compared to HCs.

**TABLE 3:** Comparison of oculomotor performance between sALS and bALS.

Task	Feature	sALS (*n* = 31)	bALS (*n* = 15)	*p* value		
				sALS vs. HC	bALS vs. HC	sALS vs. bALS
Prosaccade	Accuracy, %	96.63 ± 11.92	96.29 ± 7.87	0.392	0.302	0.909
	Latency, ms	242.08 ± 42.00	250.52 ± 47.54	0.645	0.377	0.563
	Reaction time, ms	311.43 (273.39, 363.16)	301.34 (267.93, 337.84)	0.448	0.062	0.556
	Velocity, °/s	233.42 ± 82.17	216.42 ± 90.05	0.741	0.421	0.542
Antiaccade	Accuracy, %	44.44 (28.57, 57.14)	22.92 (0.00, 53.13)	0.772	0.170	0.119
	Latency, ms	338.28 ± 47.79	353.91 ± 81.89	0.068	0.071	0.502
	Reaction time, ms	357.20 ± 68.49	358.06 ± 44.92	**0.016**	**0.013**	0.960
	Velocity, °/s	224.90 ± 72.66	220.65 ± 105.85	0.443	0.531	0.890
	Uncorrected error rate, %	0.00 (0.00, 22.22)	6.25 (0.00, 72.9)	0.874	0.642	0.480
	Error correction reaction time, ms	289.86 ± 159.02	311.30 ± 171.71	0.287	0.223	0.688
Predictive saccade	Accuracy, %	89.03 ± 20.02	82.12 ± 16.97	**0.008**	**0.002**	0.256
	Latency, ms	319.56 (289.43, 349.72)	350.29 (329.18, 370.91)	0.059	0.953	0.142
	Number of predications	0.00 (0.00, 0.75)	1.00 (0.00, 2.00)	0.202	0.055	0.069
Smooth pursuit	Initial time, ms	455.47 (286.83, 565.78)	530.85 (299.81, 681.34)	0.615	0.595	0.700
	Tracking velocity, °/s	21.30 (15.48, 28.21)	22.87 (9.45, 33.12)	0.278	0.460	0.219
	Tracking acceleration, °/s^2^	30.78 (20.81, 74.95)	55.54 (21.26, 48.91)	0.530	0.616	0.682
	Number of deviations	15.00 (9.75, 26.75)	10.00 (8.00, 28.00)	0.689	0.555	0.547
	Deviation (> 4°), °	5.19 (4.84, 5.70)	5.29 (4.89, 5.94)	0.495	0.637	0.700
	Total deviation (> 4°), °	79.94 (28.24, 142.48)	63.83 (47.24, 145.66)	0.524	0.791	0.885
Fixation	Accuracy, %	83.66 ± 24.27	78.59 ± 27.12	0.977	0.536	0.544
	Number of deviations (> 4°)	5.50 (2.00, 13.75)	7.00 (2.00, 38.00)	0.808	0.860	0.699
	Number of deviations (> 2°)	19.00 (4.75, 41.50)	16.00 (3.00, 27.00)	0.315	0.101	0.392
	Total deviation (> 4°), °	35.00 (17.04, 85.05)	43.52 (16.59, 210.66)	0.802	0.791	0.580
	Total deviation duration, ms	659.96 (385.54, 1955.46)	1119.20 (299.55, 2418.83)	0.518	0.814	0.904

Abbreviations: bALS, ALS patients with bulbar‐onset; HC, healthy control; sALS, ALS patients with spinal‐onset ALS. Bold values indicate statistically significant results.

### 3.3 Comparison Between ALS Patients With and Without Bulbar Involvement

Patients with bulbar involvement demonstrated significantly worse performance in predictive saccade accuracy compared to those without bulbar involvement (77.53 ± 26.66% vs. 96.01 ± 4.92%, *T* = 3.860, p < 0.001) (Table 4). Additionally, patients with bulbar involvement showed longer initial time in the smooth pursuit task (647.43 [402.14, 760.64] ms vs. 452.43 [131.62, 598.20] ms, *U* = 161.00, *p* = 0.037). In comparison to HCs, the reaction times in anti‐saccade were significantly prolonged in both subgroups (*T* = 2.702, *p* = 0.010 and *T* = 2.189, *p* = 0.013, respectively), along with a notable reduction in accuracy during predictive saccade tasks (*T* = 4.750, p < 0.001 and *T* = 2.243, *p* = 0.043, respectively). Patients with bulbar involvement also demonstrated a longer latency in anti‐saccade (346.12 ± 54.82 ms vs. 310.46 ± 61.75 ms, *T* = 2.071, *p* = 0.044) and initial time in the smooth pursuit task compared to HCs (647.43 [402.14, 760.64] ms vs. 455.47 [286.83, 565.78] ms, *U* = 166.00, *p* = 0.037).

**TABLE 4:** Comparison of oculomotor performance ALS with and without bulbar involvement.

Task	Feature	Bulbar involvement (*n* = 23)	No bulbar involvement (*n* = 23)	*p* value		
				Bulbar ALS vs. HC	No‐bulbar ALS vs. HC	Bulbar vs. No‐bulbar
Prosaccade	Accuracy, %	93.47 ± 14.95	99.57 ± 2.14	0.123	0.284	0.065
	Latency, ms	250.11 ± 47.65	239.57 ± 39.16	0.335	0.796	0.417
	Reaction time, ms	311.43 (260.07, 363.16)	301.34 (271.29, 322.01)	0.307	0.057	0.716
	Velocity, °/s	232.41 ± 92.99	223.33 ± 73.50	0.739	0.477	0.715
Antiaccade	Accuracy, %	40.97 (0.00, 58.48)	42.86 (28.57, 57.14)	0.312	0.715	0.410
	Latency, ms	346.12 ± 54.82	340.62 ± 83.09	**0.044**	0.169	0.792
	Reaction time, ms	363.82 ± 66.31	351.13 ± 55.80	**0.010**	**0.034**	0.486
	Velocity, °/s	208.28 ± 95.57	238.76 ± 69.84	0.208	0.927	0.224
	Uncorrected error rate, %	10.56 (0.00, 72.92)	0.00 (0.00, 20.00)	0.481	0.587	0.171
	Error correction reaction time, ms	298.63 ± 128.86	295.07 ± 188.30	0.204	0.308	0.941
Predictive saccade	Accuracy, %	77.53 ± 26.66	96.01 ± 4.92	**0.001**	**0.042**	**0.003**
	Latency, ms	340.12 (309.85, 369.97)	330.45 (229.21, 360.78)	0.151	0.031	0.345
	Number of predications	0.50 (0.00, 1.50)	1.00 (0.00, 1.75)	0.385	0.157	0.755
Smooth pursuit	Initial time, ms	647.43 (402.14, 760.64)	452.43 (131.62, 598.20)	**0.048**	0.606	**0.037**
	Tracking velocity, °/s	22.87 (15.85, 35.3)	20.05 (15.17, 25.34)	0.423	0.820	0.414
	Tracking acceleration, °/s^2^	53.96 (20.98, 197.87)	30.34 (23.06, 58.38)	0.598	0.180	0.196
	Number of deviations	13.00 (9.00, 42.00)	15.00 (9.25, 19.75)	0.939	0.265	0.547
	Deviation (> 4°), °	5.27 (4.89, 5.91)	5.16 (4.81, 5.82)	0.568	0.892	0.683
	Total deviation (> 4°), °	67.54 (52.89, 220.02)	79.94 (51.25, 103.39)	0.991	0.212	0.482
Fixation	Accuracy, %	76.89 ± 28.02	87.19 ± 21.40	0.337	0.517	0.169
	Number of deviations (> 4°)	8.00 (3.00, 38.00)	5.00 (2.00, 11.25)	0.552	0.438	0.206
	Number of deviations (> 2°)	9.00 (4.00, 41.00)	20.5 (4.00, 37.25)	0.119	0.363	0.625
	Total deviation (> 4°), °	54.36 (18.14, 210.66)	32.82 (14.33, 64.34)	0.546	0.467	0.134
	Total deviation duration, ms	1119.20 (377.17, 3572.41)	632.11 (371.66, 1075.85)	0.886	0.247	0.340

Abbreviations: ALS, amyotrophic lateral sclerosis; HC, healthy control. Bold values indicate statistically significant results.

### 3.4 Comparison Between Patients With and Without UMN Involvement

Patients with UMN involvement showed significantly longer prosaccade latency (257.32 ± 46.85 ms vs. 209.48 ± 32.00 ms, *T* = 3.267, *p* = 0.002), longer anti‐saccade latency (357.32 ± 71.13 ms vs. 303.85 ± 68.13 ms, *T* = 2.310, *p* = 0.032), and lower predictive saccade accuracy (83.86 ± 21.97% vs. 95.03 ± 4.70%, *T* = 2.789, *p* < 0.008) compared to those without UMN involvement (Table 5). In the fixation task, although the accuracy was slightly lower, patients with UMN involvement showed fewer deviations and a smaller total deviation compared to patients without UMN involvement (*U* = 108.00, *p* = 0.020; *U* = 106.50, *p* = 0.017; *U* = 118.0, *p* = 0.040). Compared to HCs, both subgroups exhibited a significant lengthening of the reaction times in the anti‐saccade task (*T* = 2.580, *p* = 0.013; *T* = 2.037, *p* = 0.050), accompanied by a marked decline in accuracy during the predictive saccade tasks (*T* = 4.083, *p* < 0.001; *T* = 3.020, *p* = 0.009). Patients with UMN involvement also displayed a longer latency in anti‐saccade (357.32 ± 71.13 ms vs. 310.46 ± 61.75 ms, *T* = 2.570, *p* = 0.013).

**TABLE 5:** Comparison of oculomotor performance ALS with and without UMN involvement.

Task	Feature	UMN involvement (*n* = 34)	No UMN involvement (*n* = 12)	*p* value		
				UMN ALS vs. HC	No UMN ALS vs. HC	UMN vs. No UMN
Prosaccade	Accuracy, %	96.84 ± 11.38	95.63 ± 8.25	0.406	0.256	0.697
	Latency, ms	257.32 ± 46.85	209.48 ± 32.00	0.113	0.064	**0.002**
	Reaction time, ms	313.23 (277.79, 345.20)	276.33 (262.35, 333.55)	0.234	0.137	0.236
	Velocity, °/s	220.72 ± 76.38	248.12 ± 102.93	0.396	0.867	0.412
Anti‐saccade	Accuracy, %	44.44 (17.36, 64.59)	28.57 (0.00, 50.00)	0.723	0.179	0.226
	Latency, ms	357.32 ± 71.13	303.85 ± 68.13	**0.013**	0.773	**0.032**
	Reaction time, ms	356.68 ± 61.69	356.91 ± 62.80	**0.013**	**0.050**	0.971
	Velocity, °/s	233.26 ± 79.23	195.93 ± 95.87	0.721	0.176	0.243
	Uncorrected error rate, %	10.00 (0.00, 23.61)	0.00 (0.00, 48.41)	0.789	0.790	0.639
	Error correction reaction time, ms	302.37 ± 165.28	281.22 ± 148.89	0.184	0.468	0.685
Predictive saccade	Accuracy, %	83.86 ± 21.97	95.03 ± 4.70	**< 0.001**	**0.009**	**0.008**
	Latency, ms	329.93 (299.05, 359.64)	350.81 (319.72, 380.33)	0.413	0.633	0.659
	Number of predications	1.00 (0.00, 1.00)	0.00 (0.00, 0.00)	0.134	0.734	0.885
Smooth pursuit	Initial time, ms	521.37 (303.42, 741.15)	530.85 (297.83, 653.86)	0.449	0.548	0.909
	Tracking velocity, °/s	23.34 (16.07, 29.53)	20.92 (15.00, 30.59)	0.809	0.278	0.409
	Tracking acceleration, °/s^2^	34.16 (20.00, 103.01)	32.00 (30.00, 168.46)	0.577	0.959	0.534
	Number of deviations	13.00 (7.50, 24.00)	19.00 (10.50, 38.75)	0.282	0.694	0.291
	Deviation (> 4°), °	5.29 (4.83, 5.95)	5.23 (4.88, 5.43)	0.635	0.771	0.395
	Total deviation (> 4°), °	67.54 (46.77, 128.51)	92.36 (60.71, 199.24)	0.338	0.932	0.409
Fixation	Accuracy, %	80.48 ± 27.95	86.47 ± 11.91	0.612	0.547	0.316
	Number of deviations (> 4°)	5.00 (2.00, 9.00)	11.50 (6.75, 34.50)	0.472	0.221	**0.020**
	Number of deviations (> 2°)	14.00 (3.50, 28.00)	34.00 (12.25, 53.75)	0.062	0.461	**0.017**
	Total deviation (> 4°), °	30.29 (15.07, 59.94)	70.16 (39.95, 185.44)	0.532	0.263	**0.040**
	Total deviation duration, ms	621.29 (377.19, 2047.05)	1064.30 (393.86, 2332.74)	0.521	0.851	0.695

Abbreviations: ALS, amyotrophic lateral sclerosis; HC, healthy control; UMN, upper motor neuron. Bold values indicate statistically significant results.

### 3.5 Correlation With Clinical Data and Cognitive Functions

The accuracy of the prosaccade task demonstrated a positive correlation with MMSE and FAB scores (Pearson's *r* = 0.807, *p* < 0.001; Pearson's *r* = 0.771, *p* = 0.001). After adjusting for age of onset, level of education, disease duration, ALSFRS‐R score, and UMN score, these correlations were no longer statistically significant (*B* = 0.402, *p* = 0.226; *B* = 0.575, *p* = 0.378). The number of deviations and total deviation in smooth pursuit were negatively correlated with FAB scores (rho = −0.730, *p* = 0.002; rho = −0.745, *p* = 0.001). Following logarithmic transformation of the dependent variable, multiple linear regression analysis also revealed no statistical significance (*B* = −0.312, *p* = 0.449; *B* = −0.274, *p* = 0.495). No other eye movement metrics were associated with cognitive function or clinical data (disease duration, UMN scores, ALSFRS‐R total score, ALSFRS‐R bulbar score, and disease progression rate).

## 4 Discussion

ALS patients exhibit various ocular abnormalities. Saccadic dysfunctions include reduced velocity, increased latency, and saccade hypometria in reflexive saccades (Rojas et al. 2020). Volitional saccades are further impaired, with anti‐saccades showing prolonged latencies, higher error rates, and lower correction rates, while delayed and memory‐guided saccades demonstrate premature reactions and prolonged latencies (Rojas et al. 2020). Smooth pursuit is characterized by reduced pursuit gains, increased catch‐up saccades, and abnormal cogwheeling (Rojas et al. 2020). The most common fixation abnormality is an increased frequency of saccadic intrusions (Donaghy et al. 2011). Our results do not fully align with those of previous studies, as ALS patients showed a general trend of poorer performance across all eye movement tasks, with significant statistical differences observed only in response times during anti‐saccade tasks and accuracy during predictive saccade tasks compared to HCs. The discrepancy might be due to the relatively small sample size of our study; however, from another perspective, it highlights the high sensitivity of these two parameters in detecting oculomotor abnormalities in ALS patients.

The performance of an anti‐saccade involves the engagement of the posterior parietal cortex for target localization and eye movement initiation, the activation of the FEF, and the integrative capabilities of the prefrontal cortex. While latency pertains to the initial detection and preparation phase of eye movement, reaction time covers the entire process—from the target's appearance to the eyes reaching the target—thus, reaction time provides a comprehensive measure that reflects not only the subject's response speed but also the efficiency of the eye movement execution. The prolonged anti‐saccade response time in ALS and across its phenotypes suggests impaired saccade initiation and reduced speed due to frontal lobe dysfunction. However, it should be noted that, although the task demonstrates high sensitivity as a relatively crude or basic assessment for detecting frontal lobe function in patients with neurological or psychiatric disorders, its specificity is low, meaning it can also yield positive results in patients without frontal lobe dysfunction (Levy et al. 2004).

This study represents a pioneering effort to investigate the predictive saccade paradigm in patients with ALS. The saccade stimulus alternated between two fixed positions at regular intervals, making its movement fully predictable. Participants were instructed to shift their gaze to the newly appearing saccade stimulus as quickly and accurately as possible. Predictive saccades primarily recruit brain areas involved in timing and are associated with the default mode network (DMN), rather than the oculomotor structures typically active during reactive saccades, such as FEF and supplementary eye fields (Lee et al. 2016). The crus I region of the cerebellum also plays a central role in predictive saccades by supporting the timing mechanisms necessary for sensorimotor synchronization (Lee et al. 2016; McDowell et al. 2008). The prominent reduced accuracy in predictive saccade tasks in ALS suggests damage to the DMN and/or the cerebellum, consistent with fMRI studies showing decreased DMN connectivity (Agosta et al. 2013; Trojsi et al. 2015; Tedeschi et al. 2012) and altered connectivity in both motor and extra‐motor regions of the cerebellum in ALS patients (Xuan et al. 2024; Bede et al. 2021). As the DMN is involved in internally oriented tasks such as self‐referential thinking and memory retrieval, its impairment might reflect deeper cognitive challenges underlying the observed oculomotor issues.

In this study, while bALS patients exhibited numerically greater oculomotor impairments compared to sALS patients, the differences between these subgroups did not reach statistical significance. When stratified by bulbar involvement—irrespective of initial disease presentation—ALS patients with bulbar pathology demonstrated distinct deficits, including reduced accuracy in predictive saccades and prolonged initial response times during smooth pursuit tasks. This observation aligns with the hypothesis that the anatomical distribution of neurodegeneration, rather than the pattern of symptom onset, may be the primary determinant of oculomotor dysfunction in ALS (Guo et al. 2022). The lack of significant differences between bALS and sALS subgroups could stem from the progressive dissemination of pathology, which may obscure initial phenotypic distinctions as the disease advances. By the time oculomotor assessments are conducted, widespread corticofugal degeneration in both subtypes might homogenize their oculomotor profiles. This interpretation is supported by neuropathological studies showing that pTDP‐43 pathology in ALS spreads sequentially along axonal pathways, allowing the identification of four neuropathological stages and highlighting consistent brainstem involvement regardless of the onset type (Brettschneider et al. 2013).

Notably, patients with bulbar involvement exhibited increased anti‐saccade latency compared to HCs, whereas those without bulbar involvement did not. This difference may stem from variations in the functional reserve of oculomotor networks and fronto‐brainstem connectivity between the two groups. Premotor burst neurons in the midbrain and pons may be disproportionately affected in patients with bulbar involvement due to their proximity to brainstem regions vulnerable to early TDP‐43 pathology, combined with disrupted fronto‐pontine projections, ultimately resulting in saccade initiation abnormalities. Conversely, spinal‐onset patients might initially preserve these circuits, delaying the emergence of overt oculomotor deficits until later stages. This is supported by the imaging findings that show widespread grey and white matter changes in bALS compared to sALS (Cardenas‐Blanco et al. 2014; Prell et al. 2013; Christidi et al. 2018; Kim et al. 2017). Longitudinal studies tracking oculomotor changes alongside neuroimaging biomarkers (e.g., pontine atrophy on MRI) are needed to validate this hypothesis.

Our results indicated that ALS patients with UMN involvement exhibited poorer performance on prosaccade, anti‐saccade, and predictive saccade tasks compared to those without UMN involvement, yet they unexpectedly performed better on fixation deviation tasks, and no correlation was found between UMN scores and eye movement parameters. In the study by Poletti et al. (2021), oculomotor abnormalities often appear to be associated with phenotypes characterized by prominent UMN involvement, including primary lateral sclerosis (PLS) and predominant UMN, compared to classic ALS and predominantly LMN diseases. However, they used bedside examination methods and did not include fixation dysfunction. Proudfoot et al. (2015) also observed that anti‐saccades were more impaired in PLS compared to the typical performance seen in ALS. The better performance of patients without UMN involvement in the fixation task may be attributed to two main factors. First, a small sample size could lead to instability in statistical analysis and an incomplete representation of true differences, affecting the reliability and generalizability of the findings. Second, the neural mechanisms most closely associated with successful fixation are thought to involve omnipause neurons located in the pontine region and the superior colliculus (Krauzlis et al. 2017). Consequently, brainstem pathology may have a stronger correlation with fixation abnormalities. However, this study did not stratify UMN involvement into predominantly motor cortex or brainstem regions, which limits the specificity of the conclusions that can be drawn. It is suggested that future research apply standardized quantitative MRI assessments to evaluate the distribution and extent of UMN involvement (Nitert et al. 2022), while also including patients with PLS as a control group, to provide a more comprehensive evaluation of the impact of UMN lesions on eye movements.

Unlike previous studies, our findings revealed no significant correlation between oculomotor metrics and cognitive tests or clinical data. From a technical perspective, this discrepancy could stem from methodological differences, such as a smaller sample size or lack of stratification, which may have reduced the statistical power to detect associations. Variability in the tools and protocols used to assess oculomotor and cognitive functions, including differences in task complexity and eye‐tracking system sensitivity, could also contribute. Our findings may further reflect bias due to recruiting less severely affected patients, thereby limiting the range of deficits observed. From the standpoint of underlying mechanisms, it is also possible that oculomotor dysfunction and cognitive decline progress along distinct or partially overlapping pathways, as prior studies have suggested slower declines in oculomotor function compared to other motor or cognitive domains (Proudfoot et al. 2015). Some patients may compensate for eye movement function through remaining neural networks (such as the basal ganglia–thalamic pathway) (Lynch and Tian 2006), thereby masking the correlation with cognitive scores. Additionally, whether eye‐tracking can serve as a surrogate for cognitive deficits in ALS remains uncertain, as it is unclear whether oculomotor dysfunction leads to poor performance on eye‐tracking‐based cognitive tests (false negatives) or whether preserved function conceals underlying deficits. Moreover, a cross‐sectional approach like ours may not fully capture the dynamic relationship between oculomotor parameters and cognitive or clinical outcomes. Future longitudinal studies involving larger and more diverse cohorts are essential to clarify these complex relationships and establish the role of oculomotor metrics as reliable biomarkers in ALS.

This study has several limitations that should be noted. While we previously discussed the small sample size and cross‐sectional design—which may limit the generalizability of the findings—there are additional methodological constraints that warrant attention. First, bulbar involvement in this study was determined solely based on clinical symptoms, without distinguishing between bulbar and corticobulbar involvement. Patients with only bulbar UMN signs or bulbar EMG abnormalities but no corresponding clinical symptoms were not classified as having bulbar involvement. These criteria may underestimate the complexity of bulbar and corticobulbar pathology in ALS, and more detailed subgroup classification in future, larger cohorts could help to more accurately assess the impact of these factors on oculomotor function. Second, we did not employ neurophysiological examinations such as motor evoked potentials (MEP) to evaluate UMN involvement, which may have led to an underestimation of the prevalence or severity of UMN dysfunction in our cohort. Lastly, another critical factor is the limitation of the EyeKnow system used for oculomotor testing: its relatively low sampling frequency may result in missed detection of subtle eye movement abnormalities. Collectively, these factors highlight the need for future longitudinal studies with larger sample sizes, more comprehensive subgrouping, and advanced neurophysiological and oculomotor assessment tools to validate these findings and further elucidate the interplay between motor, cognitive, and anatomical changes in ALS.

## 5 Conclusion

In summary, this study underscores the significant impact of bulbar and UMN involvement on oculomotor parameters in ALS, revealing distinct patterns of dysfunction among different phenotypes. The findings indicate that oculomotor metrics possess the potential to act as sensitive indicators of ALS‐related pathophysiology; however, their effectiveness as standalone biomarkers requires validation. Future research should focus on longitudinal studies with larger cohorts to better understand the dynamic interplay of motor, cognitive, and anatomical progression in ALS, thus facilitating the development of targeted interventions and improving patient outcomes.

## Author Contributions

**Dongchao Shen**: conceptualization, methodology, software, data curation, investigation, formal analysis, funding acquisition, writing – original draft. **Anfeng Liu**: methodology, software. **Xunzhe Yang**: data curation, investigation. **Qing Liu**: methodology, writing – review and editing, supervision. **Mingsheng Liu**: writing – review and editing, supervision, resources. **nLiying Cui**: writing – review and editing, supervision, resources.

## Ethical approval

The study was approved by the Research Ethics Committee of Peking Union Medical College Hospital (I‐23PJ324).

## Consent

Written informed consent was obtained from all participants.

## Conflicts of Interest

The authors declare no conflicts of interest.

## Peer Review

The peer review history for this article is available at https://publons.com/publon/10.1002/brb3.70906.

## Supporting information




**Supplementary Materials**: brb370906‐sup‐0001‐SuppMat.docx

## Data Availability

The data that support the findings of this study are available from the corresponding author upon reasonable request.
