# Supplementary material for: The Impact of Bulbar and Upper Motor Neuron Involvement on Oculomotor Movement in Amyotrophic Lateral Sclerosis
Source: Brain Behav. 2025 Oct 23;15(10):e70906. doi: 10.1002/brb3.70906 (PMC12550129; doi:10.1002/brb3.70906)
Supplement: Supplementary file 1 — Supplementary Materials: brb370906‐sup‐0001‐SuppMat.docx [file BRB3-15-e70906-s001.docx]

**Eye movement paradigm description:**

1. Prosaccade Task:

- Description: A green target dot was presented at the center of the display and randomly shifted ±15° horizontally and vertically away from the center.

- Participants were asked to promptly and accurately direct their gaze towards the target dot.

- Parameters Analyzed:

- Accuracy (%): The percentage of successful saccades out of the total number of saccade tests conducted.
- Latency (ms): The time taken for a saccade to begin after the target appeared.
- Reaction Time (ms): The average duration between the target onset and the moment a correctly executed saccade arrived at the target spot, considering all saccades.
- Velocity (°/s): The angular speed of the eye movement, calculated by measuring the angular displacement during the interval from saccade onset to saccade offset.

2. Anti-saccade Task:

- Description: Similar to the prosaccade task, a green target dot was presented, but participants were asked to execute a saccade to the opposite position of the target dot as soon as possible.

- Parameters Analyzed:

- Accuracy (%): The proportion of successful and direct saccades aimed at the position in the opposite direction, relative to the total number of antisaccade tests performed.
- Latency (ms): The time duration between the onset of the target and the initiation of the corresponding saccade.
- Reaction Time (ms): The period from the target onset to the completion of an accurate saccade that successfully reached the target spot.
- Error Correction Rate (%): The ratio of corrective saccades executed from the initial spot to the opposite position, compared to the total number of uninhibited saccades directed towards the target spot.
- Uncorrected Error Rate (%): (100 - accuracy rate) * (100 - error correction rate) / 100
- Error Correction Reaction Time (ms): The duration from the onset of the corrective saccade to its completion, as it reached the opposite position.
- Velocity (°/s): The angular speed of the eye movement, calculated by measuring the angular displacement during the interval from saccade onset to saccade offset.

3. Predictive Saccade Task:

- Description: The saccade stimulus alternated between two fixed positions at regular intervals, making its movement fully predictable. Participants were instructed to shift their gaze to the newly appearing saccade stimulus as quickly and accurately as possible.

- Parameters Analyzed:

- Accuracy (%): The percentage of trials in which participants successfully directed their gaze to the target position.
- Latency (ms): The time interval between the target’s appearance and the initiation of the saccade. Notably, latency may be negative in this task if a predictive saccade occurs before the visual stimulus appears.
- Number of Predictive Saccades (times): where participants initiated an endogenous saccade in anticipation of the target’s appearance before it was visually presented.

4. Smooth Pursuit Task:

- Description: A green dot moved along a sinusoidal trajectory with a horizontal/vertical/ circular amplitude of 20° and a frequency of 0.2 Hz.

- Participants were asked to continuously track the sinusoidal movement of the target dot.

- Parameters Analyzed:

- Initial Time (ms): The duration taken from the initiation of the moving spot until the eye successfully tracked and locked onto it.
- Tracking Velocity (°/s): The angular velocity at which the eye followed the moving spot during the tracking period.
- Tracking Acceleration (°/s²): The angular rate of change in eye movement speed during the tracking period.
- Number of Deviation (times): The total count of instances where the eye deviated by more than 4° from the moving spot throughout the entire task.
- Deviation (>4°) (°): The average degree of deviation observed in each instance of offset behavior.
- Total Deviation (>4°) (°): The cumulative degree of deviation from the moving spot across the entire task.

5. Fixation Task:

- Description: A stationary green target dot was presented and participants were asked to fixate at the target dot stably as soon as possible.

- Parameters Analyzed:

- Accuracy Rate (%): The proportion of time spent fixating on the spot relative to the total duration of the entire fixation task.
- Number of Deviation (>4°) (times): The total count of instances where the eye deviated by more than 4° from the moving spot throughout the fixation task.
- Number of Deviation (>2°) (times): The total count of instances where the eye exhibited deviations exceeding 2° from the moving spot during fixation.
- Total Deviation (>4°) (°): The same definition as in the smooth pursuit task.
- Total Deviation Duration (ms): The cumulative time duration during which the eye deviated from the moving spot by more than 4°.
